# When “virtual” works and when it doesn’t: A survey of physician and patient experiences with virtual care during the COVID-19 pandemic

**DOI:** 10.1177/20552076241258390

**Published:** 2024-06-04

**Authors:** Jennifer M. Hensel, Jocelyne Lemoine, Shay-Lee Bolton, Essence Perera, Megan Arpin, Jitender Sareen, Mandana Modirrousta

**Affiliations:** 1 Department of Psychiatry, 8664 University of Manitoba, Winnipeg, Manitoba, Canada; 2 Departments of Community Health Sciences and Psychiatry, 8664 University of Manitoba, Winnipeg, Manitoba, Canada

**Keywords:** Telemedicine, patient experiences, virtual care, COVID-19

## Abstract

**Objective:**

To assess the experience of virtual care among both patients and physicians across a range of clinical scenarios during the COVID-19 pandemic.

**Methods:**

A web-based survey was disseminated to patients and physicians through a variety of media and healthcare communications from May 2020 to July 2021. Demographic details and attitudes across a range of virtual care domains were collected. Quantitative responses were analyzed descriptively. Open-text responses were gathered to contrast when a virtual visit was superior or inferior to an in-person one, and a thematic content analysis was used.

**Results:**

There were 197 patients and 93 physician respondents, representing a range of demographic and practice characteristics. Patients noted several benefits of virtual care and felt it should continue to be available. Physicians felt they could do a lot of their care virtually. Common themes related to the superiority of virtual care were for “quick” visits, reviewing test results, chronic disease monitoring, and medication needs. Virtual care was less ideal when a physical exam was needed, and was not perceived as a good fit for an individual's cultural, language, or emotional needs. Certain conditions were identified as both ideal and non-ideal for the virtual format (e.g. mental healthcare).

**Discussion:**

Certain situations are more amenable to virtual care with personal preferences among both patients and physicians. Future priorities should ensure that virtual care is effective across the range of clinical situations in which it may be used and that both virtual and in-person options are equally available to those who want them.

Internationally, the COVID-19 pandemic in 2020 sparked the rapid virtualization of healthcare as a result of public health orders to control the spread of SARS-CoV-2.^1–4^ The pandemic necessitated virtual forms of care delivery to ensure the continued health of the population; now virtual care is persisting as a mainstay of the health system given the recognition of its benefits for both providers and patients.^5^ Ongoing questions remain, however, related to how virtual care should be delivered and funded, and the degree to which quality care can be achieved and ensured.^6^ An accepted definition of virtual care is the use of “any forms of communication or information technologies with the aim of facilitating or maximizing the quality and effectiveness of patient care.”^7^ Virtual care, of course, is not a new concept; telemedicine has been available to deliver care to rural areas for some time,^8^ and the use of videoconferencing and app-enabled remote communication between patients and providers was rising pre-pandemic.^9^ During the pandemic, however, many healthcare jurisdictions dropped existing regulations and authorized healthcare workers with no previous training to provide virtual care to their patients. Of note, while proven video-based forms of communication were utilized by those who had existing infrastructure to do so when the pandemic started, the vast majority of virtual care during this rapid shift was delivered by telephone.^1,10^ The use of telephone by physicians for comprehensive assessment and management was relatively uncommon prior to the onset of the pandemic. In sum, the pandemic created an abrupt shift in practice compared to the usual path to change in healthcare, pushing the “majority” and “laggers” to rapidly become adopters^11,12^ with limited evidence to guide this transition.

Several studies have examined the experiences and perceptions of patients and providers during this rapid transition to virtual care enabling several systematic reviews to be published.^13–15^ These reviews have illustrated general satisfaction among patients with the quality of virtual care modalities (app, voice call, video call, or email/messaging) used to connect with their providers,^13^ and a variety of advantages and disadvantages have been identified.^14,15^ Advantages and benefits of virtual care have included continuity of care, increased convenience, increased accessibility, and decreased risk of SARS-CoV-2 infection.^14,15^ Issues and disadvantages of virtual care have included decreased non-verbal communication, lack of in-person interaction, and reduced personal connection during visits, as well as lack of access to technology and low technological literacy^14,15^ which can have negative consequences for health equity—in particular, those who live in poverty, lack access to digital technology or struggle with digital literacy.^16^ Recent large studies among general practitioners^17^ and nurse-led clinics^18^ noted additional benefits and challenges of virtual care delivery from the perspective of the providers. Li et al.^17^ identified benefits for patients around quality of care (efficiency and patient-centeredness), benefits to healthcare providers (work from home, control over work schedule), and benefits to healthcare systems (acceleration of virtual care adoption in primary care settings). In a study of nurses, Barton et al.^18^ found that the providers thought that in-person visits were inferior to virtual visits for quality care, patient-provider connection, effective communication, seeing the patients’ problems, and visiting with patients for the right amount of time. On the other hand, providers felt that virtual visits were superior to in-person visits in terms of accessing patient records, scheduling follow-up visits, and prompt follow-ups.^18^ While published reviews indicate a general consensus among patients and providers regarding the advantages and disadvantages of virtual care, understanding the nuances of when virtual care is most appropriate remains an area needing further study.^10,19^ There have been studies examining the application of virtual care for a limited range of clinical conditions and visit types including asthma self-management support,^20^ mental health services,^21–23^ cancer care,^24^ and telehealth for genetic counseling.^25^ Furthermore, most studies have not examined both patient and provider perspectives.^20,21,24^ Most healthcare systems have seen the opportunity for virtual care and are now looking at how to operationalize and integrate it going forward. For a period of time, the pandemic necessitated virtual options for nearly all non-emergent healthcare interactions, but this was a unique situation, and a better understanding of the appropriateness of virtual care across different clinical conditions, clinical interactions, and virtual care modalities is needed to inform the delivery of appropriately funded, high-quality care going forward.^26^

This article reports the results of a large survey of patients and physicians in one Canadian province and contributes to the growing body of literature aimed at understanding the appropriateness of virtual care to inform its place in the future of healthcare delivery. Both patients and physicians receiving and delivering care in a range of clinical settings were asked about their comfort with virtual care for different presenting problems and the most useful and least useful scenarios for its use. A better understanding of the ways in which virtual care is best utilized will better equip healthcare systems to improve the implementation and utilization of this treatment modality.

## Methods

### Study design and setting

This was a mixed methods study, concurrently collecting quantitative and qualitative data.^27^ This study consisted of a cross-sectional, anonymous, web-based survey of physicians and patients who delivered and received healthcare in Manitoba, Canada during the COVID-19 pandemic. Manitoba is a centrally located province in Canada which has universal healthcare meaning that all physician services are offered free of charge to all residents with valid healthcare coverage. Any patient who received care with a virtual visit during the study period and any practicing physician providing virtual care were eligible to participate. All survey data were collected anonymously for a period of 14 months between 1 May 2020 and 1 July 2021. During this time, recommendations were in place for virtual to be the preferred method of interaction when possible. Manitoba had a provincial telehealth program pre-pandemic, allowing physicians to provide remote consultation to rural healthcare settings through a traditional telehealth suite but no other virtual care infrastructure. As most physicians work in a fee-for-service compensation model, the government introduced virtual visit tariffs during the pandemic for most types of care which were equivalent to in-person fees. The first wave of COVID-19 infections was relatively contained in Manitoba, but the second and third waves saw a much higher rate of spread and associated hospitalization and fatalities. These waves occurred during this study, the second from September 2020 to February 2021 and the third starting in April 2021 and ending just after the study's completion in August 2021. Public health mandated gathering restrictions and recommended stay-at-home orders during these periods.

### Survey design

This study consisted of two separate surveys: one for patients and one for physicians. The survey questions were initially designed by one of the principal investigators (MM) and were sent to other investigators for feedback, regarding the form and the type of the questions. Factors that were considered when designing the questions included usability, clarity, and length, whether they could with reasonable chance cover the attitude of survey participants toward virtual care, and comprehensiveness of the questions. After the final consensus and the ethics approval, the survey was sent to a total of 20 volunteers (10 healthcare workers and 10 patients) for testing. These responses were not included in the final study sample. Suggestions that the investigators felt improved the survey were incorporated and a revised ethics application was submitted for final approval.

Surveys were hosted on Survey Monkey, consisting of both close- and open-ended, free-text questions (see Supplemental Table S1 for patient and provider survey questions). Close-ended questions were primarily presented in multiple-choice and matrix-preference formats. Informed consent was obtained from all individual participants included in the study.

#### Physician survey

The physician survey included questions on the provider demographics, including their age, gender, the setting in which they provided care (e.g. hospital inpatient, hospital outpatient, community, and emergency/crisis), patient ages, and the type of care they provided for patients (consultation (i.e. initial assessment of a new problem), follow-up (i.e. repeat visit related to a previously assessed problem), group therapy). Additional questions focused on prior and current experience with virtual care delivery, opinions regarding the quality of care related to virtual care, and whether virtual care is appropriate for certain patient groups (new, existing, and chronic disease patients). Among the questions included were ones regarding the benefits of virtual care on provider practice and sustainability. These included the type of virtual care used (telephone, personal videoconferencing (e.g. Zoom), telehealth provided through a telehealth suite at a healthcare center), text-based communication (email or text), the number of weekly visits done through virtual care, the availability of support for implementation, and overall job satisfaction since implementing virtual care.

Open-ended questions explored provider opinions on which circumstances might lead them to prefer virtual to in-person visits and vice versa and allowed respondents to express any additional comments they have about virtual care going forward.

#### Patient survey

The patient survey asked for demographic information (age, gender, and urban/rural residency), setting and type of provider seen, mode of virtual care visit (telephone, video, and text-based communication), and prior experience with virtual care. Patients were asked about their comfort with virtual care modalities, technology in general, privacy concerns, and whether they believe virtual care should be available after the COVID-19 pandemic. The authors used a 5-point Likert scale to assess the comfort level of using technology to engage in virtual care, with various methods of virtual care including telephone, telehealth, videoconferencing, and text-based communication (email or text) (1—very uncomfortable; 2—somewhat uncomfortable; 3—neither comfortable nor uncomfortable; 4—somewhat comfortable; and 5—very comfortable). To assess the level of importance of having a virtual option for care, the following Likert values were used: 1 = not at all important; 2—not so important; 3—somewhat important; 4—very important; and 5—extremely important. To assess the level of difficulty using the technology required to participate in virtual care a Likert scale was used with the following values: 1—very easy; 2—easy; 3—neither easy nor difficult; 4—difficult; and 5—very difficult). And to assess patients’ level of concern that privacy may be breached during virtual care the Likert values were 1—not at all; 2—not so concerned; 3—somewhat concerned; 4—very concerned; and 5—extremely concerned. Similar to the physician survey, patients were also asked to respond, with open text, when they felt a virtual care visit was superior to an in-person visit and correspondingly when it was felt to be inferior.

### Survey dissemination

Links to the surveys were distributed to physicians through e-mails sent to members of relevant professional groups and postings on affiliated websites (e.g. Doctors Manitoba, The College of Physicians & Surgeons of Manitoba). Patients were informed about the survey by their physician or their office either through a short note added to the bottom of the digital invitation they received for their virtual visit or verbally at the end of their virtual care visit. Patients who were interested and wished to participate were directed to the SurveyMonkey link through which they could access and complete the survey. Information about the study that included the survey links was disseminated through newspapers and TV news announcements. Participation in the study was voluntary and anonymous.

### Data analysis

Quantitative data were analyzed in Statistical Package for the Social Sciences (SPSS) version 28.^28^ For physician responses, we descriptively summarized the respondent characteristics and calculated frequencies for the categorical responses. Patient respondent characteristics were similarly summarized, and means were calculated for the Likert Scale data.

Open-text responses to questions in both surveys that captured perspectives about the superiority or inferiority of a virtual visit relative to an in-person visit were analyzed inductively using qualitative thematic content analysis.^29^ Initially, data were reviewed and coded by a single researcher for presentation to local policymakers. To enhance reliability, a second coder later independently reviewed the initial codes, and the two team members met over several meetings to consolidate any additional codes identified and any discrepancies (i.e. inter-coder agreement).^27^ Reliability was also enhanced by keeping a code book and sharing it between coders throughout the coding process.^30^ Codes were quantified by frequency of occurrence, using the total number of respondents who offered a text-based response to the question as the denominator to calculate a proportion. Responses to open-ended questions in the healthcare provider survey about perceived benefits to practice and “other comments” were also reviewed for content regarding potential benefits and sustainability factors for the future of virtual care which were summarized as a list.

## Results

### Physicians

A total of 93 physician responses were included. Among the physicians included, the mean age was 46.8 years (SD  =  11.81). The physician sample was 50.5% female, 47.3% male, and 2.2% unspecified (n  =  93). Among physician respondents, 34 (36.6%) worked in mental health and 59 (63.4%) worked in physical specialties. Sixty-three physician respondents (67.7%) worked in hospital settings (14% inpatient, 50.5% outpatient, and 3.2% emergency/crisis), and the rest in community-based practices (32.3%). Most physicians were doing consultations (78.5%) and follow-up visits (63.4%) with a small proportion offering group-based treatment (9.7%). Most physicians were seeing patients between the ages of 18–65 (70%)*.* 76.3% of physicians report using Electronic Medical Records.

Table 1 summarizes the physicians’ experience with virtual care prior to and during the early phases of the COVID-19 pandemic. Prior to the COVID-19 pandemic, 38.8% of physicians had delivered healthcare in a virtual setting, inclusive of telephone, videoconference, and traditional telehealth suites. In contrast, 71% of physicians reported providing more than 50% of their care through virtual means since the onset of the COVID-19 pandemic. The uptake of virtual care delivery was expedited due to the pandemic, with nearly 85% of physicians learning to implement virtual care provision in < 2 weeks, with 43% of physicians reporting that they did not receive any support in implementing virtual care. Although some physicians may have had prior experience with telehealth, they newly adopted telephone and/or personal videoconferencing appointments. Forty-seven percent noted a decrease in job satisfaction during this time. The vast majority of physicians (80%) felt that virtual care should continue to be publicly funded under the province's healthcare plan going forward. While 44% of physicians noted that their length of visits was shorter, 40% noted that they required more preparation or effort.

**Table 1.** Physician experience with virtual care delivery prior to and during the pandemic.

	All specialties n = 93	
	*n*	% (95% CI)
**Prior to the pandemic, % of practice was virtual care**		
0%	57	61.3 (51.6–71.0)
1%–25%	34	36.6 (26.9–46.2)
26%–100%	2	2.2 (0.0–3.2)
**Current % of practice that is virtual care**		
0%	0	0
1%–25%	12	12.9 (6.5–19.4)
26%–50%	15	16.1 (8.6–23.7)
51%–75%	21	22.6 (14.0–32.3)
76%–100%	45	48.4 (8.6–43.0)
**Average number of virtual visits per week**		
1–20	57	61.3 (51.6–71.0)
21–40	23	24.7 (17.2–33.3)
41–100	9	9.7 (4.3–15.1)
> 100	4	4.3 (1.1–8.6)
**Virtual care type used (check all that apply)**		
Telephone	92	98.9 (96.8–100)
Videoconferencing	44	47.3 (37.6–57.0)
Telehealth	26	28.0 (19.4–37.6)
Text-based communication	10	10.8 (4.3–17.2)
**Number of patients seen in a day**		
Fewer patients	40	43.5 (33.7–53.3)
About the same	41	44.6 (34.8–55.4)
More patients	11	12.0 (5.4–18.5)
**Job satisfaction since COVID-19**		
Decreased	39	47.0 (37.3–57.8)
Stayed the same	33	39.8 (28.9–50.6)
Increased	11	13.3 (7.2–20.5)
**Believe virtual care should be funded publicly**		
Yes	80	87.0 (79.3–93.5)
No	3	3.3 (0.0–7.6)
Not sure	9	9.8 (4.3–16.3)

**Figure 1.**
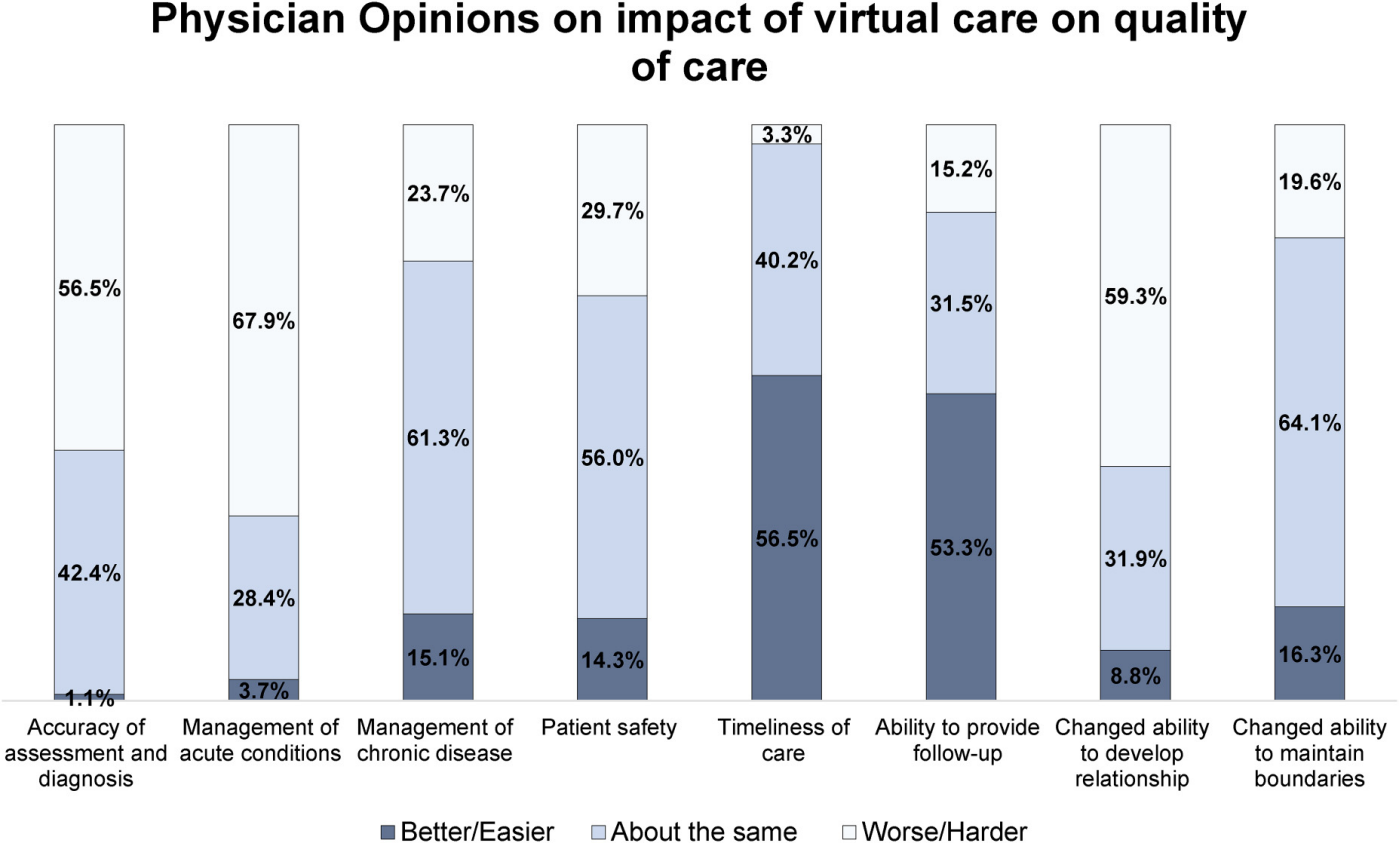
Physician opinions on the impact of virtual care on quality of care.

In Figure 1, the physician samples’ opinions on the quality of care affected by virtual care are presented. Physicians generally found that their timeliness of care was better, with only 3.3% reporting that the shift to virtual care delivery worsened their timeliness of care. Many of the physicians reported their management of acute conditions (67.9%), ability to develop relationships with patients (59.3%), and ability to accurately assess and diagnose patients (56.5%) had gotten worse/harder. The majority of physicians found that there were no notable differences in their quality of care concerning the management of chronic disease, patient safety, and ability to maintain boundaries.

**Figure 2.**
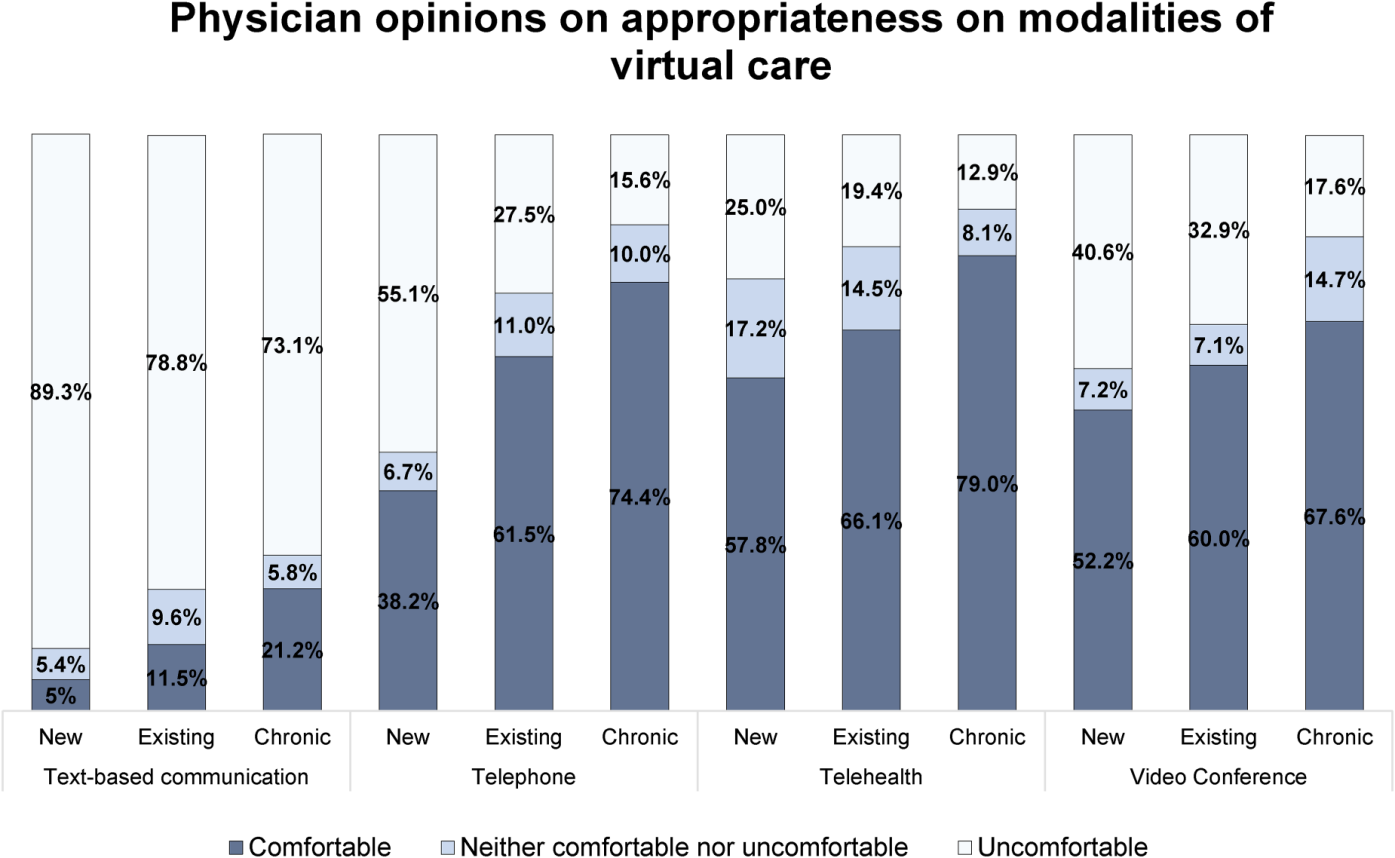
Physician opinions on the appropriateness of modalities of virtual care.

Figure 2 illustrates the appropriateness of virtual care modalities for certain groups of patients (new patients, existing patients, and chronic disease patients). Physicians reported their comfort with video conferencing, telephone, telehealth, and email and text-based communication with new, existing, and chronic disease patients. Overall, physicians reported more comfort with chronic disease patients, with the highest level of comfort using telehealth at 79%. Physicians reported similar levels of relative discomfort with text-based communication with new (89.3%), existing (78.8%), and chronic disease patients (73.1%).

### Patients

A total of 197 patient responses were included. One additional patient initiated the survey but did not provide information about their visit and so was not included in the analysis. The mean age of patients included is 54.8 years (SD = 11.81). Self-reported gender includes 57 males, 136 females, 1 other, and 2 prefer not to say. A majority of patients resided in urban areas (78%), whereas the remaining were in rural areas (22%). 94.9% of patients received virtual care during the COVID-19 pandemic while outside of a healthcare facility (e.g. at home). The majority (70.6%) did not use videoconferencing platforms. Of those who used videoconferencing platforms in virtual care, most used Zoom 18.8% (N  =  37). Patient comfort levels with the types of technology required for virtual care were between “neither comfortable nor uncomfortable” and “somewhat comfortable.” With mean Likert values for each modality as follows: video conference was 3.5, telehealth was 3.9, telephone was 3.3, and text-based communication was 3.3 (Likert scale: 1  =  very uncomfortable, 2  =  somewhat uncomfortable, 3  =  neither comfortable or uncomfortable, 4  =  somewhat comfortable, and 5  =  very comfortable).

Table 2 describes virtual care details reported by patients. Most patients saw a GP/Family doctor (80%) outside of a healthcare facility (95%) for follow-up care/care for a chronic health concern (56%) via telephone (86%). The majority of patients felt it was important to have a virtual option for care (73%), they found that the technology was easy to use (73%) and liked that it's convenient and easy (69%) and saves time (61%) and doesn't require transportation (66%). 74.6% of patients agree that publicly funded virtual care should be available to them after the COVID-19 pandemic.

**Table 2.** The patient reported receipt of virtual care and related experiences.

	*n*	Frequency % (95% CI)
**Virtual care visits prior to the pandemic**		
No	181	91.9 (87.8–95.4)
Yes	16	8.1 (4.6–12.2)
**What service(s) have you received via virtual care during the COVID-19 pandemic (indicate as many as apply)?**		
Family doctor	157	79.7 (73.8–84.7)
Specialist doctor	106	53.8 (46.9–60.8)
**Methods of communication used for virtual care visits**		
Telephone	170	86.3 (81.6–91.0)
Videoconferencing	45	22.8 (16.8–28.9)
Text-based communication	24	12.2 (7.7–17.4)
Telehealth	2	1.0 (0–2.7)
Other	1	0.5 (0–1.6)
**What is your preference regarding virtual or in-person healthcare visits?**		
I would prefer my healthcare to be virtual visits wherever possible (e.g. when I don’t need a physical examination)	113	57.4 (49.0–63.0)
I prefer in-person visits	64	32.5 (26.2–39.5)
I don’t have a strong preference either way	20	10.2 (5.9–14.4)
**What do you like most about virtual care?**		
It's convenient and easy	135	68.5 (62.0–74.9)
No transportation is required	130	66.0 (58.9–72.4)
It saves me time	121	61.4 (54.8–68.8)
It means I did not have to go to a clinic and potentially expose myself to COVID-19	121	61.4 (54.3–68.1)
It allows for more frequent visits with my doctor	28	14.2 (9.5–20.0)
I do not like anything about virtual care	20	10.2 (6.2–14.5)
It allows for better care provision	11	5.6 (2.5–9.1)
**What do you like least about virtual care?**		
I feel that I am not assessed properly	60	30.5 (23.6–36.8)
I dislike not seeing the healthcare worker in person	56	28.4 (21.9–35.0)
I have concerns about a breach of privacy	21	10.7 (6.4–15.3)
I find it difficult to use the technology	8	4.1 (1.5–6.9)
I don’t have access to the required technology	5	2.5 (0.5–5.0)
There is nothing I dislike about virtual care	75	38.1 (30.8–44.9)
**To what extent do you feel that publicly funded virtual care should be available to you once the COVID-19 pandemic is over?**		
Agree	147	74.6 (68.0–80.7)
Neither agree nor disagree	32	16.2 (11.2–21.3)
Disagree	18	9.1 (5.6–13.7)
**To what extent do you think it is important to have a virtual option for care?**		
Important	143	72.6 (66.5–78.7)
Somewhat important	30	15.2 (10.2–20.3)
Not important	24	12.2 (7.6–16.8)

### Qualitative data

In both the physician and patient surveys, a range of situations where virtual visits were superior and inferior to in-person care were identified (see Supplemental Table S2 for physician results and Supplemental Table S3 for patient results). There were themes that arose exclusively in one or the other comparison, whereas there were also themes that were present in both implying that for some of the situations where a virtual visit was perceived as superior, it was perceived by other respondents as inferior in the same situation. Many themes in both comparisons were common between the physicians and patient respondents.

#### A virtual visit is superior to an in-person visit

In both respondent groups, themes were categorized as relating to the type of visit, visits for particular health conditions, and medication-related needs. Patient respondents additionally talked about overcoming barriers through virtual care, whereas physicians discussed patient considerations which included barriers to attending the clinic but also other factors that can be enhanced or overcome with virtual care, as well as other efficiencies in care delivery.

#### Visit type

For both physician and patient respondents, virtual visits were considered superior to in-person visits when the visits were “quick” (*n*  =  5/80, 6.3% and *n*  =  4/178, 2.2%), or for follow-ups of known issues (*n*  =  27/80, 33.8% and *n*  =  21/178, 11.8%, respectively). Visits for the purposes of initial assessment (*n*  =  6/178, 3.4%), general advice (*n*  =  9/178, 5.1%), or to “check-in” (*n*  =  5/178, 2.8%) were described by patients as amenable to the virtual format. Physicians described preferring virtual visits when no physical exams or interventions were required (*n*  =  8/80, 10.0%) or for the purposes of education and counseling (*n*  =  2/80, 2.5%), whereas patients talked about care planning and co-ordination visits (*n*  =  4/178, 2.2%). A substantial proportion of both physicians (*n*  =  18/80, 22.5%) and patients (*n*  =  39/178, 21.9%) mentioned virtual visits as ideal for the purposes of ordering or reviewing test results. A number of patients reported that virtual visits were helpful for “all” of their care (*n*  =  9/80, 11.3%) or “none” of their care (*n*  =  2/178, 1.1%).

#### Disease type

In terms of the nature of the condition for which care was being sought, both patients and physicians preferred virtual visits for chronic diseases that require ongoing management (*n*  =  21/178, 11.8% and *n*  =  10/80, 12.5%) as well as minor and uncomplicated conditions (n  =  2/178, 1.1% and *n*  =  3/80, 3.8%, respectively). Physicians also thought that stable and predictable diseases (*n*  =  2/80, 2.5%) were suitable for virtual care. Additionally, some patients mentioned particular conditions such as mental health (n  =  6/178, 3.4%), cognitive disorders (n  =  4/178, 2.2%), and perinatal care (n  =  2/178, 1.1%) were suitable for virtual care.

#### Medication

Addressing medication needs was noted by both patients (*n*  =  50/178, 28.1%) and physicians (*n*  =  3/80, 3.8%) as a good fit for virtual visits. Examples of such medication needs included medication review and dose changes, prescription refills, and initiation of some medications such as antibiotics.

#### Overcoming barriers and patient considerations

Patients described a variety of barriers to attending at the clinic that were overcome by virtual care. Virtual care addressed barriers including travel (*n*  =  6/178, 3.4%), waiting time (*n*  =  3/178, 1.7%), work obligations (*n*  =  1/178, 0.6%), and mobility issues due to physical limitations (*n*  =  1/178, 0.6%). Similarly, physicians (*n*  =  6/80, 7.5%) described the benefit of virtual care to overcome mobility issues for some patients. Additional patient considerations were identified in the physician responses including childcare responsibilities (*n*  =  2/80, 2.5%), patients who are elderly (*n*  =  2/80, 2.5%), individuals who frequently do not show up for scheduled clinic appointments (*n*  =  4/80, 5%), patients with transportation issues (*n*  =  5/80, 6.3%), patients who live far away (*n*  =  14/80, 17.5%; rural or remote areas), and those who might be at personal health risk by attending in the clinic (*n*  =  1/80, 1.3%; e.g. individuals who are immunocompromised).

#### Efficiencies

Physicians also described the efficiencies of virtual visits that may enhance care delivery. Physicians described virtual visits as an alternative in the context of lack of access to the clinic (*n*  =  3/80, 3.8%), facilitating very rapid access (*n*  =  1/80, 1.3%), facilitating the involvement of patient supports (*n*  =  3/80, 3.8%), and offering the option for a blended model of in-person and virtual care (*n*  =  2/80, 2.5%).

#### An in-person visit is superior to a virtual visit

In both respondent groups, themes were categorized into the type of visit, visits for particular health conditions, and physical exam/procedure needs or requirements. Patient respondents additionally talked about inefficiencies related to virtual visits, whereas physicians discussed patient factors considered for in-person visits.

#### Visit type

For both physician and patient respondents, in-person visits were considered superior to virtual visits when patients were new to physicians (*n*  =  28/80, 35.0% and n  =  3/155, 1.9%), patients presented with new problems (*n*  =  4/80, 5.0% and *n*  =  14/155, 9.0%) or complex issues (*n*  =  4/80, 5.0% and *n*  =  2/155, 1.3%), and patients presented for post-operative visits (*n*  =  1/80, 1.3% and *n*  =  1/155, 0.6%, respectively). Some physician (*n*  =  9/80, 11.3%) and patient (*n*  =  3/155, 1.9%) respondents thought that in-person visits were superior to virtual visits for “all” care needs. Other patient respondents (*n*  =  19/155, 12.3%) reported that in-person visits were superior to virtual visits for “none” of their care needs, meaning virtual visits were adequate in all scenarios. Physician respondents described preferring in-person visits for the purposes of acute/emergency care (*n*  =  5/80, 6.3%), administering interventions to patients (*n*  =  2/80, 2.5%), and delivering bad news to patients (*n*  =  1/80, 1.3%), whereas patients talked about specialist consults (*n*  =  3/155, 1.9%) and physical complaints (*n*  =  2/155, 1.3%).

#### Disease type

In terms of the nature of the condition for which care was being sought, physicians preferred in-person visits for prenatal care (*n*  =  3/80, 3.8%), counseling for mental disorders or mental health and psychotherapy (*n*  =  9/80, 11.3%), and health conditions with unstable or changing symptoms which required monitoring or assessing for deterioration (*n*  =  4/80, 5.0%). Patients preferred in-person visits for infection (*n*  =  4/155, 2.6%), digestive/stomach issues (*n*  =  4/155, 2.6%), cancer care (*n*  =  1/155, 0.6%), and other various ailments (*n*  =  3/155, 1.9%; i.e. mental health, pain, and asthma) as well as conditions or diseases that are neurological (*n*  =  2/155, 1.3%) and dermatological (*n*  =  2/155, 1.3%). Visits that required a physical exam or procedure were noted by both patients (*n*  *=*  34/155, 22.1%) and physicians (*n*  =  34/80; 42.5%) as a better fit for in-person visits.

#### Inefficiencies

Patients described some inefficiencies related to virtual visits. Virtual visits were noted as problematic when additional in-person visits were still required (*n*  =  8/155, 5.2%), patients struggled to describe their own symptoms to physicians and they couldn’t “show” things to the physicians (*n*  =  4/155, 2.6%; e.g. over the phone), technology made communication between patients and physicians challenging (*n*  =  3/155, 1.9%), they replaced in-person visits even though patients preferred them (*n*  =  2/155, 1.3%), the physicians didn’t show up to the appointment or ended the visit early (*n*  =  2/155, 1.3%), and care was delayed (*n*  =  2/155, 1.3%; i.e. starting treatment, completing tests, and obtaining necessary paperwork).

#### Patient considerations

Physicians described patient factors that they considered when holding patient visits in-person. Physicians preferred in-person visits when relationship building with patients was a priority (*n*  =  6/80, 7.5%); patients did not have access to technology to connect virtually (*n*  =  3/80, 3.8%); patients preferred in-person visits (*n*  =  1/80, 1.3%); patients’ home environments were disruptive (*n*  =  1/80, 1.3%); and patient communication-, language-, or culture-related factors were present (*n*  =  3/80, 3.8%).

### Additional potential benefits for physicians and future sustainability factors

Physicians noted benefits of virtual care that were COVID-specific such as offering access to care in the context of no alternative due to public health restrictions, permitting continued access during quarantine (of both providers and patients), and reducing the risk of infection to self and others. Virtual care was seen as possibly offering more flexibility (physicians more able to offer after-hours support), increased physician availability, and addressed access barriers for individuals living in rural/remote areas and those with other barriers to attending at the office. Physicians also noted benefits to their own workflow and capacity including the opportunity for remote work, efficiencies related to not having a physical waiting room, reduced no-show appointments, and reduced stress. Being able to see the home environment over videoconferencing was seen as adding clinical value for some individuals. However, physicians also noted limitations and challenges with virtual care including more stress in some cases, technology difficulties, reduced quality of assessment, permitting more patient-driven interaction which could be both too little or too much, and the possibility that the benefits of coming to a clinic appointment (e.g. for behavioral activation and socialization) are lost. Remuneration was described as needing attention for the future sustainability of virtual care, along with technology infrastructure and support.

## Discussion

This study collected the experiences and perspectives of both physicians and patients delivering and receiving virtual care during the early parts of the COVID-19 pandemic. This was a time of rapid transition and adaptation with both groups shifting to a new model of care. While many studies have reported on the experiences of these groups during this unusual period of rapid change,^13–15,31^ few have utilized both quantitative and qualitative data and directly compared the experiences of both to elicit commonalities and differences with regards to when virtual care is ideal and when it may not be. These data can help to inform the future of virtual care delivery post-pandemic and contribute to the growing foundation that will support future research. In this study, we found high levels of acceptability for virtual care among physicians and patients, particularly during a time when alternatives were not as available and fear around the virus was high. The majority of virtual interactions occurred over the telephone. Physicians expressed varying levels of comfort with virtual care across different clinical scenarios, with the most comfort for existing patients and especially those requiring chronic disease management, and lower levels of comfort for new patients who were unknown to them, particularly through the use of telephone only. Patients also felt that virtual care provided a range of benefits, most commonly, convenience, reduced travel and time required, and avoidance of infection risk which was especially relevant during the pandemic. For the most part, physicians and patients agreed on the scenarios for which a virtual interaction would be superior to in person, that is those where the visit is a follow-up or check-in with a known provider, usually around a known or low complexity need. Communicating test results, providing counseling, or monitoring chronic diseases were seen as particularly amenable to the virtual format. Conditions that made in-person care more challenging such as geographical distance, travel, mobility issues, and other barriers contributed to virtual being seen as superior as it facilitated attendance. Naturally, visits, where a physical exam was required, were seen as less suitable for the virtual format, as were visits where the relationship was more of a priority or cultural and other factors were better attended to in person. While physicians noted the ability to rapidly triage as a positive, patients mentioned the fact that they often had to attend a second in-person visit when the virtual visit wasn’t sufficient. High proportions of both groups felt that virtual care had a place in the healthcare system and should continue to be publicly funded going forward.

Findings from this study are largely consistent with what has been reported elsewhere.^17,18,32^ Virtual care existed pre-pandemic but was not widely adopted and the imposition created by the pandemic necessitated everyone to quickly open up to the possibility of this as a viable mode of healthcare delivery.^17^ The positive attitudes among both physicians and patients reflect findings from work that predated the pandemic and have been found in numerous other studies done across a variety of settings and health conditions.^13^ Virtual care offers a range of benefits that increase access, convenience, and attendance. That said, there are a number of limitations to what it can achieve. Of course, the ability to conduct a physical exam is limited through a virtual interaction, particularly, when that interaction occurs over the telephone in the absence of any visual cues. Additionally, individuals continue to have personal preferences as to how they receive healthcare. Many individuals still prefer to attend in person in the absence of other barriers, as they see this as an important aspect of their assessment and relationship with the provider.^23,32,33^ Providers also may not feel confident in their ability to accurately assess, diagnose, and manage illness in the absence of visual cues.^31^ Interestingly, we saw some individuals feel that virtual was superior for certain health needs while others felt it was inferior for the same conditions (e.g. mental health and perinatal care). There is a strong individual preference when it comes to receiving healthcare that can be influenced by an individual's personal circumstance, barriers to attending in the office, and comfort with technology.^10,23^ In a comparable study that assessed public opinions about the future of virtual care in Manitoba, 85% of survey respondents expressed a preference to have in-person visits available as an option for all healthcare encounters.^10^

What does this mean for the future of virtual care in the health system? There is no question that virtual care provides options. Through virtual means we can extend services to hard-to-reach populations and increase access; however, these visits may not be equally appropriate for all situations. In some cases, virtual is just a step in the care episode, to determine the next steps, triage and screen, and so on. It can also be a useful tool to follow up on an in-person encounter, to review results, and treatment response, and provide education and counseling. Both parties may be most comfortable in the context of a pre-existing relationship or known condition,^10,32^ however, in many situations a virtual visit between a new patient and provider or for a new problem can likely achieve the desired outcome. While the telephone offers convenience and has fewer technological issues, expanding the availability and adoption of other virtual means such as videoconferencing will offer additional options that allow patients and providers to “see” each other, interpret cues, and give visual evidence of a health concern. Chen et al.^34^ reviewed the literature on telephone versus video interactions for mental health care and noted that both can be beneficial, although some use of video is likely better for both patients and providers. Advancements in other virtual tools such as wearables and modes to collect standardized patient-reported measures and images will enhance the ability of the physician to collect the required information in a reliable way to make an accurate assessment.^35^ Physicians also have preferences and comfort with particular formats of care delivery.^31^ If health systems are to adopt virtual care options as a basic option for healthcare, understanding the needs of physicians to deliver these options will be essential to making virtual care equally available to all those who desire and could benefit from it, and conversely preserving in-person care for those who do best with that format.

This study has a number of limitations. It used a convenience sample of patients and providers using virtual care in a variety of settings and for a range of healthcare needs. It was conducted early in the COVID-19 pandemic when virtual care was still very new for the majority of users and there were limited alternatives.^4^ While perspectives were collected from both physicians and patients, these respondents are not dyads so we can make general comparisons but cannot contrast for specific clinical scenarios. Furthermore, over 60% of the physician sample worked in a hospital-based setting and over one-third in mental health specifically. This reflected the setting in which the authors worked, and the channels through which the study was advertised, and little is known about the individual provider clinics that disseminated the survey. It may also reflect availability to respond. In contrast, 80% of patient respondents reported their virtual care occurring with a primary care provider. Although the survey asked about email and text communication, it should be noted that this type of communication is not currently reimbursed through fee-for-service payment models in the province. This study focuses on physician care which is publicly funded in our jurisdiction; whereas a range of healthcare disciplines may engage in virtual care delivery including nurses, psychologists, and physical therapists, many of whom will be subject to private pay which may introduce additional factors in the decision to receive virtual versus in-person care. The situations in which virtual care will work across disciplines may vary, although common themes are evident.^18^ The results are based on opinions only and do not reflect empirical evidence of the superiority or inferiority of virtual care for particular situations. Moreover, this study did not seek opinions from individuals who did not use telehealth for reasons related to personal choice or access. Additional evidence is needed to determine whether virtual care can achieve similar outcomes to in-person care, across a range of modalities (telephone vs. videoconferencing) and a range of clinical scenarios, in an equitable way.

## Conclusions

This study provides additional support for the continuation of virtual care within the healthcare system. Virtual care increases convenience and flexibility for both physicians and patients, but it does not suit every clinical situation or individual patient preference. More research is needed to directly compare virtual to in-person, especially in areas where there is less agreement on whether virtual is as good of a fit for a clinical scenario. Ensuring mechanisms are in place to support quality of care is required so that virtual can continue without compromising health outcomes or increasing disparities.

## Supplemental Material

sj-docx-1-dhj-10.1177_20552076241258390 - Supplemental material for When “virtual” works and when it doesn’t: A survey of physician and patient experiences with virtual care during the COVID-19 pandemic Supplemental material, sj-docx-1-dhj-10.1177_20552076241258390 for When “virtual” works and when it doesn’t: A survey of physician and patient experiences with virtual care during the COVID-19 pandemic by Jennifer M. Hensel, Jocelyne Lemoine, Shay-Lee Bolton, Essence Perera, Megan Arpin, Jitender Sareen and Mandana Modirrousta in DIGITAL HEALTH

sj-docx-2-dhj-10.1177_20552076241258390 - Supplemental material for When “virtual” works and when it doesn’t: A survey of physician and patient experiences with virtual care during the COVID-19 pandemic Supplemental material, sj-docx-2-dhj-10.1177_20552076241258390 for When “virtual” works and when it doesn’t: A survey of physician and patient experiences with virtual care during the COVID-19 pandemic by Jennifer M. Hensel, Jocelyne Lemoine, Shay-Lee Bolton, Essence Perera, Megan Arpin, Jitender Sareen and Mandana Modirrousta in DIGITAL HEALTH

sj-docx-3-dhj-10.1177_20552076241258390 - Supplemental material for When “virtual” works and when it doesn’t: A survey of physician and patient experiences with virtual care during the COVID-19 pandemic Supplemental material, sj-docx-3-dhj-10.1177_20552076241258390 for When “virtual” works and when it doesn’t: A survey of physician and patient experiences with virtual care during the COVID-19 pandemic by Jennifer M. Hensel, Jocelyne Lemoine, Shay-Lee Bolton, Essence Perera, Megan Arpin, Jitender Sareen and Mandana Modirrousta in DIGITAL HEALTH
