# Supplementary material for: When “virtual” works and when it doesn’t: A survey of physician and patient experiences with virtual care during the COVID-19 pandemic
Source: Digit Health. 2024 Jun 4;10:20552076241258390. doi: 10.1177/20552076241258390 (PMC11151755; doi:10.1177/20552076241258390)
Supplement: sj-docx-1-dhj-10.1177_20552076241258390 - Supplemental material for When “virtual” works and when it doesn’t: A survey of physician and patient experiences with virtual care during the COVID-19 pandemic [file sj-docx-1-dhj-10.1177_20552076241258390.docx]

*For the purposes of this survey, "virtual care" refers to a health care 'visit' between health care provider and patient in which care is provided in real-time via audio, video, or text-based communication,* ***not including calls to Health Links.***

1. To begin the survey, please select the appropriate option below, then click 'Next'
   1. I am a patient who has received virtual care
      1. *Note: all who select this option receive questions 2-23*
   2. I am a health care worker who has provided virtual care
      1. *Note: all who select this option receive questions 24-53*
2. What is your age?

_______

1. What is your gender?
   1. Male
   2. Female
   3. Other
   4. Prefer not to answer
2. Do you live in an urban or rural (population under 1,000) area?
   1. Urban
   2. Rural
3. What service(s) have you received via virtual care during the COVID-19 pandemic? (e.g. GP, specialist - please specify)
   1. Emergency care
   2. GP/family doctor
   3. Psychologist
   4. Group therapy
   5. Nurse/nurse therapist
   6. Other therapist (physical, occupational, respiratory, speech, social worker, etc.)
   7. Specialist doctor/Other (please specify) ____________(*text entry*)
4. In what settings have you received a virtual care visit during the COVID-19 pandemic?
   1. Emergency
   2. While I was admitted in hospital
   3. Through a government funded/public health institute (e.g. nursing station, health center/clinic)
   4. While I was outside of a health care facility (e.g. at home, etc.)
5. Which of the following best describes your virtual care visit(s) during the COVID-19 pandemic?
   1. Visit with a health care provider I’d never seen before
   2. Visit with a health care provider I’d seen before but for a new health concern
   3. Visit with a health care provider I’d seen before for follow-up care or care of a chronic health concern
6. What method(s) of communication have you used to for your virtual care visits during the COVID-19 pandemic? (*can select more than one*)
   1. Telephone
   2. Personal video-conferencing (e.g. Skype, Zoom, FaceTime etc.)
   3. Telehealth (going to a Shared Health location such as a nursing station, health center, hospital)
   4. E-mail or instant/text messaging
   5. Other: __________ (*text entry*)
7. Which of the following video-conferencing platforms have you used for your video virtual care visits during the COVID-19 pandemic? (*can select more than one*)
   1. Zoom
   2. Microsoft Teams
   3. Google Meet (G Suite or Hangouts)
   4. FaceTime
   5. Skype
   6. WhatsApp
   7. Doxy.me
   8. Medeo
   9. Other (please specify): __________ (*text entry*)
   10. I have not used any video-conferencing platforms for virtual care
8. How comfortable are you using each of the following technologies for virtual health care visits?

|  | Very uncomfortable | Somewhat uncomfortable | Neither comfortable nor uncomfortable | Somewhat comfortable | Very comfortable | N/A |
| --- | --- | --- | --- | --- | --- | --- |
| Telephone |  |  |  |  |  |  |
| Personal video-conferencing |  |  |  |  |  |  |
| Video-conferencing using Telehealth |  |  |  |  |  |  |
| Text-based communication |  |  |  |  |  |  |

1. For which of your health care needs have you found virtual visits to be the **most** helpful? (please state the ***type of care*** you received, not the name of the health care provider) __________ (*text entry*)
2. For which of your health care needs have you found virtual visits to be the **least** helpful? (please state the ***type of care*** you received, not the name of the health care provider) __________ (*text entry*)
3. Had you ever had a virtual care visit prior to the COVID-19 pandemic?
   1. Yes (please describe)
   2. No
4. During the COVID-19 pandemic, how important do you feel it was to have the option of a virtual rather than an in-person visit?
   1. Extremely important
   2. Very important
   3. Somewhat important
   4. Not so important
   5. Not at all important
5. How difficult have you found using the technology required to participate in virtual care?
   1. Very easy
   2. Easy
   3. Neither easy nor difficult
   4. Difficult
   5. Very difficult
6. How concerned are you that your privacy may be breached (i.e. that your personal or health information may not be secure) during virtual care?
   1. Extremely concerned
   2. Very concerned
   3. Somewhat concerned
   4. Not so concerned
   5. Not at all concerned
7. What is your preference regarding virtual or in-person health care visits?
   1. I would prefer if all of my health care was virtual visits
   2. I would prefer my health care to be virtual visits wherever possible (e.g. when I don’t need a physical examination)
   3. I don’t have a strong preference either way
   4. I prefer in-person visits
8. What do you like **most** about virtual care? (*can select more than one*)
   1. It’s convenient and easy
   2. No transportation is required
   3. It saves me time
   4. It allows for more frequent visits with my doctor
   5. It allows for better care provision
   6. It means I did not have to go to a clinic and potentially expose myself to COVID-19
   7. I do not like anything about virtual care
   8. Other benefit not listed above: ______________ (*text entry*)
9. Please describe any additional **benefits** you feel that virtual care has brought to your health care experience during the COVID-19 pandemic: ______________________________________________________________________________________________________________________________________________________________________________________________________________________________
10. What do you like **least** about virtual care? (*can select more than one*)
    1. I find it difficult to use the technology
    2. I don’t have access to the required technology
    3. I have concerns about a breach of privacy
    4. I feel that I am not assessed properly
    5. I dislike not seeing the health care worker in person
    6. There is nothing I dislike about virtual care
    7. Other problem not listed above: ______________ (*text entry*)
11. Please describe any additional **challenges** that virtual care has added to your health care experience during the COVID-19 pandemic: ______________________________________________________________________________________________________________________________________________________________________________________________________________________________
12. To what extent do you feel that publicly funded virtual care should be available to you once the COVID-19 pandemic is over?
    1. Strongly agree
    2. Agree
    3. Neither agree nor disagree
    4. Disagree
    5. Strongly disagree
13. Are there any additional comments you would like to make regarding the use of virtual care or your experience with the transition to virtual care during the COVID-19 pandemic? ______________________________________________________________________________________________________________________________________________________________________________________________________________________________
14. What type of health care provider are you?
    1. Family doctor
    2. Psychologist
    3. Nurse
    4. Physician assistant
    5. Other health care professional (physical/occupational/respiratory/speech & language therapist, psychotherapist, etc.)
    6. Specialist (please specify): __________ (*text entry box*)
15. In what setting do you primarily provide care?
    1. Hospital – inpatient
    2. Hospital – outpatient
    3. Emergency
    4. Private practice – group
    5. Private practice - individual
    6. Other public/shared health facility: ___________ (*text entry box*)
16. Which of the following best describes the type of care you typically provide? (*select all that apply*)
    1. Individual (one-on-one) consultation
    2. Individual follow-up care
    3. Small group-based care (2-5 individuals)
    4. Large group-based care (5-12 individuals)
    5. Very large group-based care (>12 individuals)
    6. Other (please describe): __________ (*text entry*)
17. What age demographic do the majority of your patients fall into?
    1. Pediatric (<18 years of age)
    2. Young adult (18-35)
    3. Middle-aged (36-65)
    4. Older adult (>65)
    5. I treat patients of all ages
18. Do you work using an Electronic Medical Records system?
    1. Yes
    2. No
19. What is your age?

_____

1. What is your gender?
   1. Male
   2. Female
   3. Other
2. What method(s) of communication have you used to conduct virtual care visits? (*can select more than one*)
   1. Telephone
   2. Personal video-conferencing (video based care to patients in their home)
   3. Video-conferencing to patients attending at a telehealth suite
   4. E-mail or instant/text messaging
   5. Other: __________ (*text entry*)
3. Which of the following personal video-conferencing platforms have you used to conduct video virtual care visits? (*can select more than one*)
   1. Zoom
   2. Microsoft Teams
   3. Google Meet (G Suite or Hangouts)
   4. FaceTime
   5. Skype
   6. WhatsApp
   7. Doxy.me
   8. Medeo
   9. I have not used any video-conferencing platforms for virtual care
   10. Other (please specify): __________ (*text entry*)
4. Prior to the COVID-19 pandemic, what percentage of your practice consisted of virtual care?
   1. 0%
   2. 1-25%
   3. 26-50%
   4. 51-75%
   5. 76-99%
   6. 100%
5. Currently, what percentage of your practice consists of virtual care?
   1. 0%
   2. 1-25%
   3. 26-50%
   4. 51-75%
   5. 76-99%
   6. 100%
6. Since the onset of the COVID-19 pandemic, on average, how many virtual care visits do you provide per week?
   1. 1-5
   2. 6-10
   3. 11-20
   4. 21-30
   5. 31-40
   6. 41-50
   7. 51-75
   8. 76-100
   9. >100
7. Did you have any previous training or experience in telemedicine or virtual care before the COVID-19 pandemic?
   1. Yes
   2. No

Considering virtual care you have provided since the onset of the COVID-19 pandemic:

1. To what extent do you feel the shift to virtual care has altered the quality of your health care delivery with regards to:

|  | Much worse | Somewhat worse | About the same as before | Somewhat better | Much better |
| --- | --- | --- | --- | --- | --- |
| Accuracy of assessment and diagnosis |  |  |  |  |  |
| Management of acute conditions |  |  |  |  |  |
| Management of chronic conditions |  |  |  |  |  |
| Patient safety |  |  |  |  |  |
| Timeliness of care |  |  |  |  |  |
| Ability to provide follow-up care |  |  |  |  |  |

1. Has the number of individuals you are been able to see in a typical work day changed?
   1. I am able to see fewer patients
   2. I see the same number of patients as before
   3. I am able to see more patients
2. Compared to the length of your typical in-person patient visit, how would you judge the length of your typical virtual visit for a similar indication?
   1. Much shorter than in-person
   2. Somewhat shorter than in-person
   3. About the same as in-person
   4. Somewhat longer than in-person
   5. Much longer than in-person
3. Compared to the amount of preparation or effort required for a typical in-person patient visit, how would you judge the preparation or effort required for your typical virtual visit?
   1. Much less preparation/effort is required for virtual visits
   2. Somewhat less preparation/effort is required for virtual visits
   3. About the same amount of preparation/effort is required for virtual visits
   4. Somewhat more preparation/effort is required for virtual visits
   5. Much more preparation/effort is required for virtual visits
4. Has the use of virtual care changed your ability to develop quality health-care relationships with your patients?
   1. Yes, it is much easier to develop good patient relationships using virtual care
   2. Yes, it is somewhat easier to develop good patient relationships using virtual care
   3. No, virtual care has not changed my ability to develop patient relationships
   4. Yes, it is somewhat harder to develop good patient relationships using virtual care
   5. Yes, it is much harder to develop good patient relationships using virtual care
5. Has the use of virtual care changed your ability to maintain appropriate boundaries with your patients (i.e. the professionality of your relationship; appropriate emotional, psychological, and physical distance; the clarity of your respective roles)?
   1. Yes, virtual care has made it much easier to maintain boundaries
   2. Yes, virtual care has made it somewhat easier to maintain boundaries
   3. No, virtual care has not changed the difficulty of maintaining boundaries
   4. Yes, virtual care has made it somewhat more difficult to maintain boundaries
   5. Yes, virtual care has made it much more difficult to maintain boundaries
6. With regards to the assessment of new patients, how comfortable are you providing virtual care using the following technologies:

|  | Very uncomfortable | Somewhat uncomfortable | Neither comfortable nor uncomfortable | Somewhat comfortable | Very comfortable | N/A |
| --- | --- | --- | --- | --- | --- | --- |
| Telephone |  |  |  |  |  |  |
| Personal video-conferencing |  |  |  |  |  |  |
| Video-conferencing using telehealth |  |  |  |  |  |  |
| Text-based communication |  |  |  |  |  |  |

1. With regards to the assessment of new problems in existing patients, how comfortable are you providing virtual care using the following technologies:

|  | Very uncomfortable | Somewhat uncomfortable | Neither comfortable nor uncomfortable | Somewhat comfortable | Very comfortable | N/A |
| --- | --- | --- | --- | --- | --- | --- |
| Telephone |  |  |  |  |  |  |
| Personal video-conferencing |  |  |  |  |  |  |
| Video-conferencing using telehealth |  |  |  |  |  |  |
| Text-based communication |  |  |  |  |  |  |

1. With regards to chronic disease management, how comfortable are you providing virtual care using the following technologies:

|  | Very uncomfortable | Somewhat uncomfortable | Neither comfortable nor uncomfortable | Somewhat comfortable | Very comfortable | N/A |
| --- | --- | --- | --- | --- | --- | --- |
| Telephone |  |  |  |  |  |  |
| Personal video-conferencing |  |  |  |  |  |  |
| Video-conferencing using telehealth |  |  |  |  |  |  |
| Text-based communication |  |  |  |  |  |  |

1. In the context of the COVID-19 pandemic, how long did it take for you to learn and become comfortable providing care virtually?
   1. Less than a week
   2. 1-2 weeks
   3. 2-4 weeks
   4. 1-2 months
   5. I still don’t feel comfortable
2. What support was provided to you regarding your implementation of virtual care? (*can select more than one*)
   1. Help from experienced colleagues
   2. Structured implementation support and management
   3. IT support
   4. Admin support
   5. Department/hospital recommended guidelines
   6. I didn’t receive any support
   7. Other: ___________ (*text entry*)
3. As a result of changes to service delivery during COVID-19, I feel that my job satisfaction has:
   1. Increased
   2. Stayed the same
   3. Decreased
4. In your opinion, should virtual care be publically funded following the COVID-19 pandemic?
   1. Yes
   2. No
   3. Not sure
5. Has the ability to see your patients in a setting outside of your office helped you in any way? ______________________________________________________________________________________________________________________________________________________________________________________________________________________________
6. Based on your experience, please describe the type of care for which you feel a virtual visit is superior to an in-person visit: ______________________________________________________________________________________________________________________________________________________________________________________________________________________________
7. Based on your experience, please describe the type of care for which you feel an in-person visit is superior to a virtual visit: ______________________________________________________________________________________________________________________________________________________________________________________________________________________________
8. Are there any additional comments you would like to make regarding the use of virtual care visits or your experience with the transition to virtual care during the COVID-19 pandemic? ______________________________________________________________________________________________________________________________________________________________________________________________________________________________
