# Supplementary material for: When “virtual” works and when it doesn’t: A survey of physician and patient experiences with virtual care during the COVID-19 pandemic
Source: Digit Health. 2024 Jun 4;10:20552076241258390. doi: 10.1177/20552076241258390 (PMC11151755; doi:10.1177/20552076241258390)
Supplement: sj-docx-2-dhj-10.1177_20552076241258390 - Supplemental material for When “virtual” works and when it doesn’t: A survey of physician and patient experiences with virtual care during the COVID-19 pandemic [file sj-docx-2-dhj-10.1177_20552076241258390.docx]

**Table 3.** Physician perceptions of when a virtual visit is superior and inferior to an in-person visit.

| **Virtual Visit is Superior** | | **In-person Visit is Superior** | |
| --- | --- | --- | --- |
| **Themes/Sub-themes** | **Examples** | **Themes/Sub-themes** | **Examples** |
| **Disease type:** depending on the nature of the patient’s disease or condition |  | **Disease type:** depending on the nature of the patient’s disease or condition |  |
| **Chronic disease:** when the patient has a chronic condition | “Follow-up of chronic but stable patients.” (P33)  “Chronic patients who are well known.” (P20) | **Unstable or changing symptoms:** monitoring for/or assessing deterioration in a health condition | “(…) change in symptoms (…).” (P37) |
| **Minor/uncomplicated**  **problem:** when the patient has a minor or uncomplicated issue | “(…) Assessment up of minor acute issue such as plantar fasciitis (…).” (P2)  “Non-complicated follow-ups (…).” (P6) | **Prenatal care:** when patient visits are for prenatal care | “(…) Prenatal care especially.” (P71) |
| **Predictable disease:** when the patient has a known condition | “Follow-up on a known condition with reasonably predictable course of the disease.” (P26)  “Follow-up assessments for stable patient with no issues or concerns with minimal exam findings and symptoms with a clear existing diagnosis.” (P34) | **Mental Health:** when patient visits are for psychotherapy, mental disorder or mental health counselling (e.g. bereavement) | “(…) psychotherapy.” (P30)  “Patients who have difficulty forming connections with others might need in-person appointment for successful psychotherapy.” (P58) |
| **Visit type:** depending on the nature of the patient’s visit |  | **Visit type:** depending on the nature of the patient’s visit |  |
| **No Intervention:** when no intervention needs to be performed by the HCP on the patient (i.e., no physical exam is required) | “f/u without intervention.” (P31)  “Long term follow up of a stable problem for which a physical exam is generally not necessary.” (P41) | **All:** in-person visits are superior to virtual visits for all forms of care | “All.” (P13) |
| **Follow-up:** when the visit is a follow-up | “Follow-up care.” (P24) | **Acute/emergency:** when the visit is for an acute or emergency issue | “Emergency.” (P3)  “Acute issues.” (P33) |
| **Test results:** when test results are reviewed with the patient | “For just reviewing results of a test.” (P68) | **New consult:** when the visit for assessing a new patient | “New consultation.” (P19)  “New assessments.” (P20) |
| **Quick:** when the visit is expected to be brief | “Brief follow-ups (…).” (P38) | **Complex issues:** when the patient has complex issues and/or multiple concerns | “(…) complex patients.” (P64)  “Complicated new patient consult (…).” (P73) |
| **Initial assessment:** when the visit involves information-gathering on certain new conditions or acute issues | “(…) for new assessments of certain types of clinical conditions (…).” (P32)  “Acute issues - to determine if an in person visit is required (…).” (P10) | **New problem:** when the patient experiences new symptoms or has a new health concern | “Generally new problems (…).” (P5) |
| **Frequent visits:** when the patient requires frequent visits | “(…) Patients require weekly or even twice weekly follow up.” (P56) | **Intervention needed:** when the visit requires an intervention | “(…) intervention-based visits.” (P31) |
| **Education/ counselling:** when the patient requires education or counselling | “(…) counseling around previous assessments (…).” (P57)  “(…) Patient education visits.” (P70) | **Delivering “Bad News”:** when the visit involves the disclosure of bad news | “(…) Visits that involve delivering bad news.” (P75) |
| **Patient Considerations:** when patients face barriers to attending in-person visits |  | **Post-Operative visit:** when the visit occurs post-operatively | “For (…) postoperative patients.” (P42) |
| **Mobility issues:** when the patient’s mobility is limited | “Where patient has limited mobility.” (P47) | **Patient considerations:** when certain patient factors are taken into consideration |  |
| **Child care issues:** when the patient is a primary caregiver for one or more children | “(…) post partum moms.” (P38)  “(…) Patients really appreciate virtual visits, eliminates child care concerns (…).” (P59) | **Communication/ language/ cultural barriers:** when issues related to patient communication, language, or culture make virtual visits a challenge | “(…) For those who have communication barriers such as cognitive impairment, severe mental health problems, or those requiring an interpreter (over the phone interpretation via relay is suboptimal) (…).” (P10)  “(…) also I prefer in-person when significant language/ cultural barrier exist.” (P82) |
| **Physical distance issues:** when the patient resides in a rural or remote area | “Stable chronic patients who live geographically far away (i.e. outside of city limits).” (P21)  “Long distance follow up.” (P29) | **Need for relationship building:** when relationship-building is a priority | “Making secure relationship for the patient since they can look you in the eye.” (P83) |
| **Transportation issues:** when transportation is a challenge for the patient | “(…) those with significant transportation difficulties such as patients with dementia (…).” (P49) | **Technology barriers:** when patients do not have access to technology to connect virtually | “Many of my patients don't have a phone and most don't have internet, so virtual visits are challenging (…).” (P52) |
| **Elderly:** when the patient is elderly | “It is more convenient. I also see older patients and some patients with visual impairments. This has made their life easier.” (P45) | **Preferences:** patient preference for in person visit | “(…) some patients are uncomfortable with the virtual set up.” (P62) |
| **Frequent no-shows:** when the patient frequently misses appointments | “Where patients would typically be avoidant, anxious, or forgetful, therefore missing appointments.” (P54)  “(…) For patients who often miss in-person appointments (…).” (P56) | **Home Environment:** Hectic or disruptive home environment | “(…) those in hectic home environments (hard to hear over the phone).” (P56) |
| **Personal health risk:** a visit to the clinic might put the individual at elevated risk; e.g. immunosuppressed | “If the patient is immunosuppressed (…) then a virtual care platform appointment is likely superior. (P61) | **Physical exam/ procedure required:** when are physical exams or procedures are needed to provide appropriate care | “A very large part of my practice involves physically examining patients to diagnose or monitor conditions which is impossible with virtual care.” (P21) |
| **Efficiencies:** when virtual visits are a reasonable alternative for the patient’s care and may enhance care delivery |  |  |  |
| **Alternative for accessing care:** when it is not possible for the patient to receive care in-person | “(…) All issues for those who do not have access to transportation.” (P10)  “For individuals truly unable to attend an in-person visit (…).” (P40) |  |  |
| **Blended model:** when a combination of in-person and virtual visits is ideal for the patient’s care | “Chronic care, intermittently. (…).” (P19)  “Following patients with well established chronic conditions in between in person visits. (…).” (P21) |  |  |
| **Involvement of supports/providers:** when it facilitates the involvement of family, other informal supports or health care providers to attend or participate in the patient’s care | “At times can remove barriers to clients and family members attending and/or participating (…).” (P51)  “(…) Case conference with multiple disciplines, including PCP.” (P60) |  |  |
| **Rapid Access:** when the visit needs to happen quickly | “When there is a need for the patient to be seen very quickly. (P63) |  |  |
| **Medication:** when the visit is regarding the patient’s medication, eg. refills, follow-up | “Medication follow up.” (P39) |  |  |
