# Supplementary material for: When “virtual” works and when it doesn’t: A survey of physician and patient experiences with virtual care during the COVID-19 pandemic
Source: Digit Health. 2024 Jun 4;10:20552076241258390. doi: 10.1177/20552076241258390 (PMC11151755; doi:10.1177/20552076241258390)
Supplement: sj-docx-3-dhj-10.1177_20552076241258390 - Supplemental material for When “virtual” works and when it doesn’t: A survey of physician and patient experiences with virtual care during the COVID-19 pandemic [file sj-docx-3-dhj-10.1177_20552076241258390.docx]

**Table 4.** Patient perceptions of when a virtual visit is superior and inferior to an in-person visit.

| **Virtual Visit is Superior** | | **In-person Visit is Superior** | |
| --- | --- | --- | --- |
| **Themes/Sub-themes** | **Examples** | **Themes/Sub-themes** | **Examples** |
| **Disease type:** depending on the nature of the patient’s disease or condition |  | **Disease type:** depending on the nature of the patient’s disease or condition |  |
| **Minor/ uncomplicated problem:** when the patient has a minor or uncomplicated issue | “Visit with GP for minor problem is helpful since I can receive care at home.” (P6) | **Neurological:** when the visit is regarding a neurological condition | “Neurology assessments.” (P190) |
| **Perinatal:** when patient is receiving care related to pregnancy | “Pregnancy.” (P37) | **Dermatological:** when the visit is regarding dermatological disease | “(…) Dermatology.” (P42) |
| **Recurring condition:** when the patient has a recurring condition | “(…) for recurring health concern.” (P96) | **Infection:** when the patient has an infection | “It was for possible inflamed or infected colon so just a phone call didn't really help.” (P57)  “(…) for ear infection.” (P96) |
| **Chronic disease:** when the patient has a chronic condition | “(…) chronic conditions. (P79) | **Digestive/ stomach issues:** when the patient has digestive or stomach issues | “Stomach issue.” (P196) |
| **Mental health:** when the patient has mental health/ psychiatric conditions | “Psychiatry.” (P85) | **Cancer care:** when the visit is regarding cancer care | “Cancer follow up.” (P90) |
| **Cognitive:** when the patient has cognitive issues | “Testing my memory with a neuropsychologist on Zoom.” (P81)  “Dementia related care. Testing while in my own home was very comfortable.” (P149) | **Other:** when visits are for other various ailments (i.e., mental health, pain, asthma) | “Pain management.” (P138)  “Asthma.” (P129) |
| **Visit type:** depending on the nature of the patient’s visit |  | **Visit type:** depending on the nature of the patient’s visit |  |
| **All:** virtual visits were superior to in-person visits for all healthcare needs | “All.” (P58) | **Specialist consult:** when the visit is a consultation with a specialist | “Specialist visit (…).” (P41) |
| **None:** virtual visits were not superior to in-person visits for any health care needs | “None.” (P55) | **Physical complaints:** when the visit is regarding a physical complaint | “Physical complaints or new issues that need to be seen in person.” (P46) |
| **Care planning:** when the patient is care planning with the HCP | “(…) discussing the issue and options via phone.” (P15)  “(…) and pre-meeting prior to procedure.” (P165) | **New problem:** when the patient experiences new symptoms or has a new health concern | “(…) When new symptoms appeared.” (P4)  “(…) new conditions.” (P43) |
| **Follow up:** when the visit is a follow up (includes general GP, post-op, post hospital care) | “Follow up care.” (P14) | **New provider:** when the physician is new to the patient | “New patient visits.” (P92) |
| **Tests:** when lab tests are requested by the HCP or test results are reviewed with the patient | “(…) reporting back about results.” (P50)  “(…) requesting tests.” (P135) | **None:** virtual visits were adequate for any type of care the patient needed | “None.” (P56) |
| **Quick:** when the visit is expected to be brief | “For a quick visit (…).” (P25)  “(…) or a quick check-in for a chronic health concern.” (P19) | **Post-Operative visit:** when the visit occurs post-operatively | “Follow up post surgery.” (P33) |
| **Check-in:** when the purpose is to check in with and/or make general requests of the HCP | “Regular check in.” (P16)  “Family [doctor] general requests.” (P42) | **All:** virtual visits were inferior to in-person visits for the type of care the patient needed | “All.”(P126) |
| **Referrals:** when the visit is regarding a referral | “(…) referrals.” (P11) | **Complex issues:** when the patient has complex issues and/or multiple concerns | “For more complicated (…) conditions.” (P43) |
| **Rapid access:** when the visit needs to happen quickly | “Quick access to a health care worker.” (P52) | **Inefficiencies:** when virtual visits are inadequate in some aspect compared to in-person visits |  |
| **Assessment/ consultation:** when the purpose of the visit is for an initial assessment, assessment, or consultation with a regular HCP or a specialist | “Assessment.” (P59)  “Initial assessment.” (P63) | **Need to describe own symptoms:** when the patient needs to describe their own symptoms | “Describing symptoms (…).” (P4) |
| **Seeking answers and advice:** when the purpose of the visit is to ask questions and/or get specific advice | “Answers to my concerns.” (P188)  “Doctor's advice (…).” (P177) | **Provider attendance issues:** when the provider does not attend the visit or ends the visit early | “The psychiatrist never showed up for the Zoom meeting.” (P13)  “(…) hung up before all questions asked.” (P88) |
| **Overcomes Barriers:** when patients face barriers to attending in-person visits that are overcome by the virtual visit |  | **Patient preference for in-person visits is not accommodated:** when the patient prefers in-person visits but they are not offered or are not possible | “Prefer face to face visits (…).” (P185) |
| **Travel-related issues:** when travel to the appointment is inconvenient or difficult for the patient | “Appointment usually 20 minutes That's over 7 hours on road driving to and from appointment to answer questions or offer any developments. A lot easier on my MH when I can stay home and do same on phone.” (P82)  “Don’t drive downtown or pay parking.” (P158) | **Communication challenges due to technology:** when technology makes communication a challenge | “It was at times more difficult to understand the physician on ZOOM.” (P149)  “Post surgery phone call. I am hard of hearing.” (P187) |
| **Waiting issues:** when attending a clinic waiting room is undesirable | “(…) and not sitting around other people.” (P76)  “(…) it cuts down on time spent in my day sitting in the waiting room for an hour for a three minute conversation.” (P25) | **Additional visit required:** when a second in-person visit is needed to re-evaluate or confirm | “Bigger concern with GP is not very helpful through virtual care since more test is required and I have to take another time to get the test instead of one visit to GP and blood test.” (P6)  “(…) The receptionist made me have a phone appointment first, then the doctor had me come in later when I knew the doctor would have to look in her ears.” (P24) |
| **Work obligations:** when taking time off work is required | “(…) It means I don't have to take a day off a work (…).” (P40) | **Phone is limiting:** when using the phone is limiting because it does not allow the patient to show something to the HCP | “Once had to have a phone [appointment] (…) but it really needed to be seen (by camera) or in person (…).” (P54)  “(…) It is difficult to discuss issues over the phone and frustrating. In person allows for better dialogue and assessment.” (P134) |
| **Mobility issues:** when the patient’s mobility is limited | “Handicapped so gives me easier access to my health care provider.” (P172) | **Delayed care:** when care is delayed | “It delayed treatment (…).” (P54)  “If there's any sort of tests that have to be done or if you need bloodwork paperwork it's not as immediate.” (P25) |
| **Medication:** when the visit is regarding the patient’s medication (includes review, refills, changes, antibiotics) | “Medicine changes.” (P120)  “Basic prescription needs for antibiotics.” (P49) | **Physical exam/ procedure:** when a physical exam or procedure is required | “Not everything can be done virtually, I do have to go in for a physical.” (P23)  “Anything that needs an in person exam (…).” (P38) |
